# Metabolic effects of the contraceptive skin patch and subdermal contraceptive implant in Mexican women: A prospective study

**DOI:** 10.1186/1742-4755-11-33

**Published:** 2014-04-26

**Authors:** Jesus Hernandez-Juarez, Ethel A Garcia-Latorre, Manuel Moreno-Hernandez, Jose Fernando Moran-Perez, Miguel Angel Rodriguez-Escobedo, Gerardo Cogque-Hernandez, Rubén Julián-Nacer, Xochitl Hernandez-Giron, Rosalia Palafox-Gomez, Irma Isordia-Salas, Abraham Majluf-Cruz

**Affiliations:** 1 Unidad de Investigacion Medica en Trombosis Hemostasia y Aterogenesis, Instituto Mexicano del Seguro Social, Mexico City, Mexico; 2 Escuela Nacional de Ciencias Biológicas, Instituto Politécnico Nacional, Mexico, D.F, Mexico; 3 Centro de Salud Urbano Tehuacan, JS No. 10, SSA, Tehuacan, Puebla, Mexico; 4 UMF 9, Instituto Mexicano del Seguro Social, Tehuacan, Puebla, Mexico; 5 UMF 30, Instituto Mexicano del Seguro Social, Tehuacan, Puebla, Mexico

**Keywords:** Contraceptive skin patch, Subdermal contraceptive implant, Metabolic effects, Metabolic changes, Contraception, Parche cutáneo anticonceptivo, Implante contraceptivo subdérmico, Efectos metabólicos, Cambios metabólicos, Contracepción

## Abstract

**Background:**

The contraceptive skin patch (CSP) accepted by the U.S. FDA in 2001 includes ethinylestradiol and norelgestromine, whereas the subdermal contraceptive implant (SCI) has etonogestrel and is also approved by the FDA. In Mexico, both are now widely used for contraception but their effects on Mexican population are unknown. The objective of the study was to evaluate if these treatments induce metabolic changes in a sample of indigenous and mestizo Mexican women.

**Methods:**

An observational, prospective, longitudinal, non-randomized study of women between 18 and 35 years of age assigned to CSP or SCI. We performed several laboratory tests: clinical chemistry, lipid profile, and liver and thyroid function tests. Also, serum levels of insulin, C-peptide, IGF-1, leptin, adiponectin, and C reactive protein were assayed.

**Results:**

Sixty-two women were enrolled, 25 used CSP (0 indigenous; 25 mestizos) and 37 used SCI (18 indigenous; 19 mestizos). Clinical symptoms were relatively more frequent in the SCI group. Thirty-four contraceptive users gained weight without other clinical significant changes. After 4 months of treatment, significant changes were found in some biochemical parameters in both treatment groups. Most were clinically irrelevant. Interestingly, the percentage of users with an abnormal atherogenic index diminished from 75% to 41.6% after follow-up.

**Conclusions:**

The CSP slightly modified the metabolic variables. Most changes were nonsignificant, whereas for SCI users changes were more evident and perhaps beneficial. Results of this attempt to evaluate the effects of contraceptives in mestizo and native-American populations show that clinical symptoms are frequent in Mexican users of CSP and SCI. Although these medications may affect some metabolic variables, these changes seem clinically irrelevant. Induction of abnormalities in other physiological pathways cannot be ruled out.

## Abstract (es)

**Résumé:**

**Introducción**

El parche cutáneo anticonceptivo (PCA), aceptado por la FDA en 2001, contiene etinilestradiol y norelgestromina mientras que el implante contraceptivo subdérmico (ICS) tiene etonogestrel y también fue aprobado por la FDA. En México, ambos son ahora ampliamente utilizados para la contracepción pero sus efectos sobre la población mexicana se desconocen. El objetivo de este estudio fue evaluar si estos tratamientos inducen cambios metabólicos en una muestra de mujeres mexicanas, indígenas y mestizas.

**Métodos**

Estudio observacional, prospectivo, longitudinal, no aleatorizado de mujeres entre 18 y 35 años de edad asignadas a PCA o a ICS. Realizamos varias pruebas de laboratorio: química clínica, perfil de lípidos y pruebas de funcionamiento hepático y tiroideo. También medimos las concentraciones de insulina, péptido C, IGF-1, leptina, adiponectina y proteína C reactiva.

**Resultados**

Sesenta y dos mujeres fueron estudiadas, 25 usuarias de PCA (0 indígenas y 25 mestizas) y 37 de ICS (18 indígenas y 19 mestizas). Los síntomas clínicos fueron relativamente más frecuentes en el grupo con ICS. Treinta y cuatro usuarias ganaron peso sin otros síntomas clínicos significativos. Después de 4 meses de tratamiento, encontramos cambios significativos en algunos parámetros bioquímicos en ambos grupos de tratamiento aunque la mayoría de estos cambios fueron clínicamente irrelevantes. De manera interesante, el porcentaje de usuarias con un índice aterogénico anormal disminuyó de 75% a 41.6% luego del periodo de seguimiento.

**Conclusiones**

El PCA modificó discretamente las variables metabólicas. La mayoría de los cambios fueron no significativos mientras que en las usuarias de ICS fueron más evidentes y hasta quizá benéficos. Los resultados de este intento de evaluar los efectos de los contraceptivos en poblaciones mestiza y americana muestran que los síntomas clínicos son frecuentes en las mujeres mexicanas usuarias de estos métodos. Aunque estos tratamientos pueden afectar algunas variables metabólicas, los cambios parecen clínicamente irrelevantes. La inducción de alteraciones en otras vías fisiopatológicas no se puede descartar.

## Background

Studying the side effects of contraceptives began with the oral contraceptive pill more than 50 years ago. At the end of the 20^th^ century, oral contraceptives were used by more than 100 million women worldwide [1]. In Mexico, 5.6% of women use oral contraceptives (1.12 million women in total) [2]. However, after the new contraceptive methods became available, the popularity for oral contraceptives dramatically decreased. Of course, side effects of the oral contraceptives were primarily responsible for the development of new hormonal contraceptive methods. Today, women may choose among several options, including the contraceptive skin patch (CSP) and the subdermal contraceptive implant (SCI).

The CSP was accepted by the U.S. Food and Drug Administration (FDA) in 2001 and was available for clinical use in 2002. The CSP contains 0.6 mg ethinylestradiol (EE) and 6.0 mg norelgestromine (NGMN), although this last dose may change depending on the commercial brand. The CSP mechanism of action is inhibiting the ovulation [3]. The treatment strategy includes changing the CSP every 7 days and then replacing the patch [4]. In each cycle, three different CSP are used. Each SP liberates almost 20 μg EE and 150 μg NGMN into the blood each day [5]. Therefore, plasma levels ranging from 25-75 pg/ml EE and from 0.6-1.2 ng/ml NGMN may be reached [6]. During the fourth week, no patch is used, allowing menstruation [7]. The risk of pregnancy is relatively low (1.24/100 users/year) (95% CI 0.19-2.33) [8]. Its use is not recommended in women weighing >90 kg [4].

On the other hand, the SCI has etonogestrel (ENG) and was approved by the FDA in 2006. Each SCI has ENG (68 mg) the active metabolite of desogestrel (DSG) [9]. ENG inhibits ovulation after reaching a serum level >90 pg/ml [10]. The ENG release rate is 40 μg/day between days one and five during the menstrual cycle [11]. Eight hours after insertion, serum levels of ENG increase up to a mean of 265 pg/ml, reaching the maximal concentration of the drug 96 h later [12]. After 1 year of insertion, ENG serum levels decrease to 196 pg/ml [12,13] and then slowly decline during the next years. The rate of pregnancy is 0.27, 0.30, and 0.38/100 women/year during the first, second and third years, respectively [14].

In Mexico both, the CSP and SCI are now widely used; however, we are unaware about most of the effects of these methods in our population. Therefore, our aim was to evaluate if SP or SCI may induce significant changes in several metabolic pathways in a sample of indigenous and mestizo Mexican women.

## Methods

### Sample selection

In this observational, prospective, longitudinal, non-randomized study, Mexican women between 18 and 35 years old were included. Neither researchers nor the physicians assigned the hormonal contraceptive treatment; each woman chose her contraceptive method. Group 1 was composed of users of CSP (6 mg NGM and 0.06 mg EE), whereas in Group 2 we included users of SCI (68 mg ENG). We included only one woman belonging to the same family. To be considered as indigenous, women had to belong to one of the several ethnic groups living throughout Mexico and must fulfill all of the following criteria: a) speaking a native language as a first language (with Spanish as a second language); b) living in an indigenous community; c) considering themselves as indigenous; and d) being carriers of blood group O Rh(O) D-positive [15]. If an interethnic indigenous mixture was demonstrated, the woman continued to be eligible. All categories of mestizo women were included. For all participants, we excluded those with a history of thrombotic events or hemophilia, family history of thrombosis or hemorrhagic diseases, requirement for any type of antithrombotic treatment, renal or liver disease, moderate-to-severe alcoholism or malnutrition, high blood pressure, known atherothrombotic disease, thyroid abnormalities, severe migraine, breast cancer, diabetes mellitus, abnormal uterine bleeding, autoimmune diseases, and current history of smoking. Participants were excluded if they received any contraceptive method during the four months before entering the study or if they were breastfeeding. Finally, we excluded women with more than one hormonal contraceptive method or those who used this kind of medication due to other medical reasons.

The protocol was accepted by the Ethics National Committee of Instituto Mexicano del Seguro Social.

### Clinical assessment

After being enrolled in the study, the following clinical variables were obtained from each woman before and after 4 months of contraceptive use: age, weight, height, abdominal circumference (AC), body mass index (BMI), and systolic (SBP) and diastolic blood pressure (DBP). The study was designed with follow-up period of 4 months because the frequency of side effects among Mexican women users of hormonal contraceptives appears precisely during this period of time.

### Sample collection

Sample collection was made from August 2011 to January 2013. We collected 22 ml of blood from each woman in two vacuum tubes without anticoagulant (Vacutainer, Beckton Dickinson, Rutherford, NJ, USA): one tube with EDTA (Vacutainer), and two tubes with 3.2% tri-sodium citrate (vol:vol = 1:9) (Vacutainer). Samples were centrifuged immediately after collection at 2,500 × g for 15 min and platelet-poor plasma and serum samples were obtained using a disposable Pasteur pipette. All aliquots were prepared using 2 ml Eppendorf tubes and immediately frozen at -70°C until processing. Both, basal samples and samples taken after 4-months of contraceptive treatment were analyzed immediately after collecting the last sample. For quality control tests we used commercially available control sera. With these results, Levey-Jennings graphs were constructed and interpreted in each running test.

### Sample size

Sample size was calculated considering the two most frequent metabolic abnormalities in our population, serum glucose (mean = 89 mg/dl; SD = 11.4 mg/dl), and triglycerides (mean 129.8; SD = 42.3). As a consequence, an abnormal serum glucose level was considered when ≥100 mg/dl and ≥170.1 mg/dl for triglycerides. For each of these variables we obtained a sample size calculating α = 0.05 and 1-β =0.90. Therefore, sample sizes were 23 and 24 women according to glucose and triglycerides, respectively. Although n = 24 for each study group was sufficient, we enrolled 50 women in each group considering those women not able to finish the study.

### Assays

We performed all assays before initiating the contraceptive method and after 4 months of use by means of commercially available diagnostic kits and using worldwide accepted techniques. We used Synchron LX 20 chemistry analyzer (Beckman Coulter, Fullerton, CA, USA) in order to assay serum levels of glucose, urea, creatinine, uric acid, alkaline phosphatase (AP), lactic dehydrogenase (LDH), serum glutamic pyruvic transaminase (SGPT), serum glutamic oxaloacetic transaminase (SGOT), gamma glutamyl transpeptidase (GGT), total bilirubin (TB), indirect bilirubin (IB), direct bilirubin (DB), total proteins (TP), albumin (Alb), globulins (Glb), triglycerides (TG), total cholesterol (TC), high-density lipoprotein cholesterol (HDL-C), low-density lipoprotein cholesterol (LDL-C), and very-low-density lipoprotein cholesterol (VLDV-C). Atherogenic index (AI) was calculated as follows: [TC (mg/dl)/HDL-C (mg/dl)]. AXSYM (Abbott Park Laboratories, Abbott Park, IL, USA) was used to evaluate serum levels of thyroid-stimulating hormone (TSH), total thyroxine, total triiodothyronine (T3), thyroid uptake, free thyroxine index, serum protein-bound iodine, anti-thyroglobulin antibodies, anti-thyroid microsomal antibodies as well as high-sensitivity C-reactive protein (hsCRP). With a Synergy HT platelet reader (Biotek Instruments, Winooski, VT, USA), we measured the serum levels of insulin, C peptide, and insulin growth factor-1 (IGF-1). Finally, an ELISA kit was used to evaluate leptin [Leptin (Sandwich) ELISA, DRG Diagnostics, Marburg, Germany], and adiponectin levels (Human Adiponectin ELISA kit, Invitrogen, Frederick, MD, USA).

### Statistical analysis

We used the Statistical Package for the Social Sciences (SPSS, v.16; SPSS Inc., Chicago, IL, USA). For the description of demographic characteristics of Mexican women and the results found of the variables analyzed, we used central tendency measures and dispersion. We used Kolmogorov Smirnov test to establish the statistical distribution of each variable. Paired t and Wilcoxon tests were used to analyze basal and post-treatment results. Student t test or Mann–Whitney U test was used in order to compare results between groups; p value <0.05 was considered significant.

### Ethics

In this study we required drawing blood, a procedure not routinely performed in users of contraceptives. Therefore, after all women made the decision to receive contraceptive treatment they were informed about the study and signed informed consent was obtained. In order to assure the confidentiality of the information, only the investigators had access to the complete data of the participants. Blinding was broken in case of significant clinical or laboratory abnormalities. The study was carried out according to national and international regulations for clinical research: Ley de General de Salud, the Helsinki Declaration, and the Code of Nuremberg.

## Results

### General data

**Figure 1:**
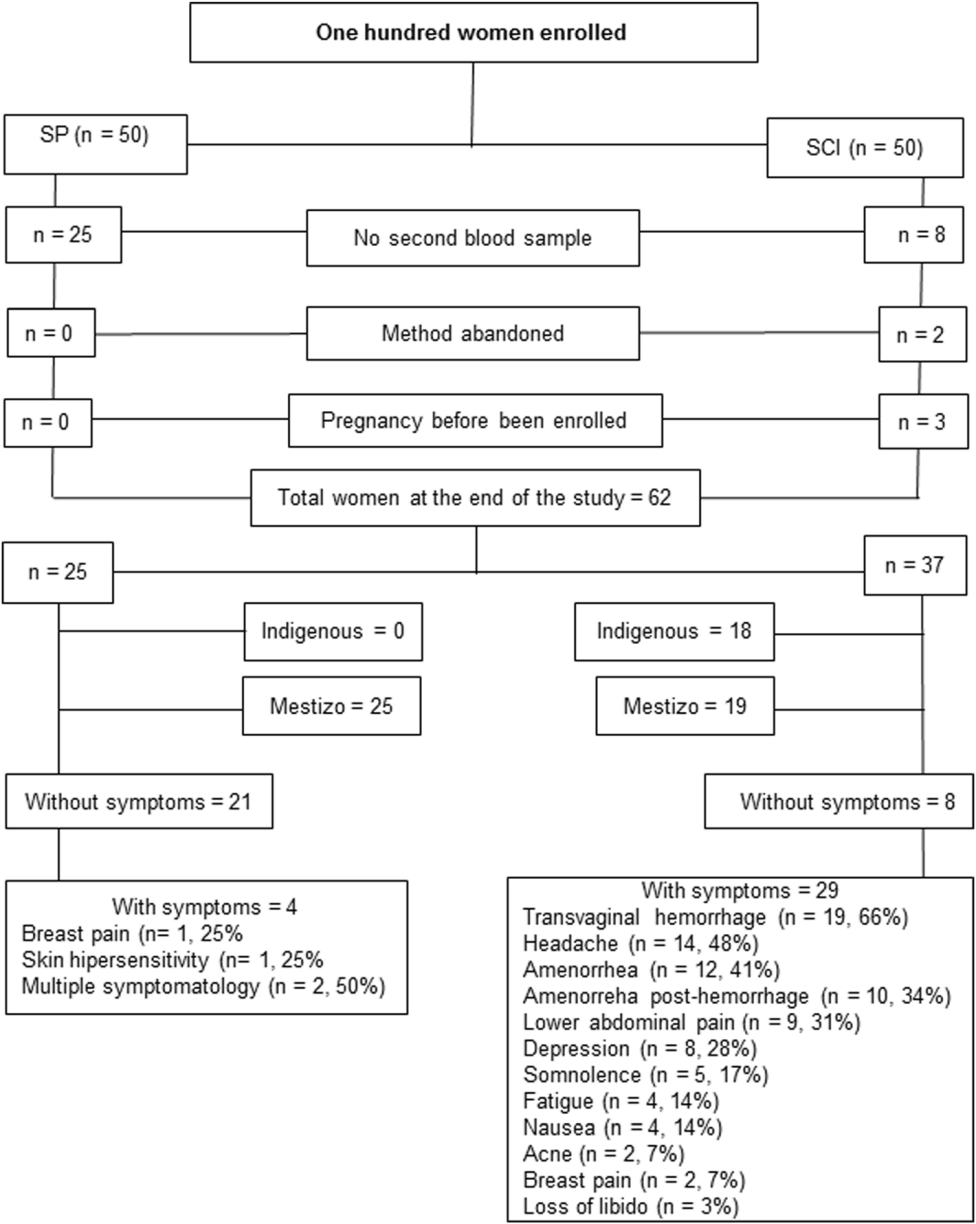
Complete study design.

We included 100 women; however, 38 women did not complete the study because they did not have the second blood sample drawn, the contraceptive method was abandoned, or because they were already pregnant when the contraceptive method was initiated (Figure 1). The main cause for study withdrawal was clinical symptomatology induced by the contraconceptive method itself. Therefore, 62 women completed the study protocol (Figure 1): 25 women were allocated to the CSP group while 37 corresponded to the SCI group. Eighteen women were indigenous and 44 were mestizo. Clinical characteristics of the whole group are shown in Table 1.

**Table 1:** Demographics of users of hormonal contraceptive treatment (n = 62)

	**CSP (n = 25)**			**SCI (n = 37)**		
**Variable**	**Basal**	**4 months**	**p**	**Basal**	**4 months**	**p**
Age (years)	23.9 (18-32)*	23.9 (18-32)	NA	28.0 (18-35)*	28.0 (18-35)	NA
Weight (kg)	59.3 (42-90)	60.9 (47-88)	0.058	57.5 (36-91)	60.0 (39-101)	0.056
Height (m)	1.6 (1.5-1.7)	1.6 (1.5-1.7)	NA	1.52 (1.38-1.64)	1.52 (1.38-1.64)	NA
BMI (kg/m^2^)	24.6 (19.2-39)	25.3 (21.5-38.1)	0.062	24.7 (16.0-38.5)	25.8 (17.3-39.9)	0.062
AC (cm)	79 (62-110)	80.5 (64.0-109)	0.057	82 (58-101)	85.1 (60-109)	0.053
SBP (mmHg)	108 (90-120)	107 (100-120)	0.588	107 (90-120)	107 (90-120)	0.980
DBP (mmHg)	71 (60-80)	70 (60-80)	0.695	68 (60-80)	69 (68-80)	0.678

Results are presented as mean (ranges). AC, abdominal circumference; BMI, body mass index; SBP, systolic blood pressure; DBP, diastolic blood pressure. *p < 0.050 between basal contraceptive skin patch (CSP) vs. basal subdermal contraceptive implant (SCI).

Clinical symptoms were relatively frequent during the study period (Figure 1). During the 4-month study period with the CSP, four users had moderate to severe symptomatology: breast pain, skin hypersensitivity, and multiple minor symptomatology. On the other hand, 29 (78%) women receiving SCI had at least one moderate to severe clinical symptom. Nineteen of these women had transvaginal hemorrhage followed by amenorrhea in ten of the women (27%). Abnormal uterine bleeding ranged from minimal and occasional bleeds up to 15-day menstrual periods. Other symptoms related to this contraceptive method are shown in Figure 1. We observed other clinical changes among the enrolled women (Table 1). For example, 13 users of the CSP and 21 of the SCI gained weight: 0.4-9 Kg and 0.4-10 Kg, respectively. However, six women (one from the CSP group and five from the SCI group) lost between 0.5 and 4 Kg. BMI and AC also increased, although these changes were not significant. No significant changes in SBP or DBP were observed. None of the women who completed the study became pregnant.

### Basal tests

For both groups no significant renal, liver, or thyroid abnormalities were identified. None of the women had diabetes but five (9.2%) had a serum glucose >100 mg/dl. Almost 30% of the women had a TG level >166 mg/dl; 66% had HDL-C <45 mg/dl; and the AI was >4.0 in 57.4% of the participants (Tables 2 and 3). Levels of insulin, C peptide, IFG-1, adiponectin, and leptin were always normal (Tables 4 and 5). AP (p = 0.040) and LDH (p = 0.035) were higher in women in the SCI treatment group. There were no other significant differences between the study groups.

**Table 2:** Metabolic variables before and after 4 months with SCI

**Variable**	**Basal**	**4 months**	**p**	**Reference values**
Glucose [mg/dl]	91.3 (80.7-116.6)	85.4 (66.0-100.2)	**0.034**	65-114
Urea [mg/dl]	21.8 (14.4-29.0)	20.7 (12.8-28.8)	0.405	15-39
Creatinine [mg/dl]	0.59 (0.4-0.7)	0.60 (0.5-0.8)	0.179	0.5-1.2
Uric acid [mg/dl]	4.5 (2.7-6.1)	4.2 (3.0-6.3)	0.418	2.6-7.2
TG [mg/dl]	136.9 (64.5-244.7)	104.1 (34.7-220.3)	**0.002**	54-214
TC [mg/dl]	163.7 (118.2-214.1)	144.6 (93.0-206.2)	**0.001**	140-220
HDL-C [mg/dl]	38.2 (20.6-60.8)	38.7 (25.0-58.4)	0.751	21-75
LDL-C [mg/dl]	98.0 (66.2-145.8)	84.7 (48.4-124.2)	**0.012**	56-155
VLDL-C [mg/dl]	27.3 (12.9-48.9)	20.8 (6.9-44.1)	**0.025**	
AI	4.54 (2.7-6.4)	3.83 (2.2-5.3)	**0.002**	<4
TB [mg/dl]	0.63 (0.2-1.2)	0.56 (0.3-1.0)	0.121	0.2-1.0
DB [mg/dl]	0.08 (0.0-0.1)	0.11 (0.1-0.2)	**0.050**	0.0-0.2
IB [mg/dl]	0.55 (0.2-1.1)	0.45 (0.2-0.8)	**0.025**	
SGOT [U/l]	25.2 (15.1-48.0)	22.3 (13.2-32.0)	0.167	15-37
SGPT [U/l]	24.9 (10.0-59.3)	21.5 (8.7-39.5)	0.081	8-35
AP [U/l]	80.2 (58.2-144.9)	75.8 (48.7-121.7)	0.093	37-110
LDH [U/l]	144 (110.8-190.0)	154.5 (115.6-195.9)	**0.005**	106-274
GGT [U/l]	21.7 (5.7-68.6)	26.9 (7.0-76.4)	**0.009**	5-24
TP [g/dl]	7.0 (6.3-7.6)	7.2 (6.8-7.8)	0.121	6.7-8.2
Alb [g/dl]	4.1 (3.7-4.6)	4.3 (3.9-4.8)	0.132	3.8-5.1
Glb [g/dl]	2.84 (2.3-3.1)	2.83 (2.4-3.3)	0.747	

Results are expressed as medians (percentiles: 2.5-97.5). Reference values (RV) for metabolic variables are showed in the fifth column. Significant p values are shown in bold. Glucose (p = 0.012), triglycerides (TG; p < 0.001), total cholesterol (TC; p < 0.001), serum glutamic oxaloacetic transaminase (SGOT; p = 0.002), serum glutamic pyruvic transaminase (SGPT; p < 0.001), alkaline phosphatase (AP; p = 0.001), total bilirubin (TB; p = 0.027), indirect bilirubin (IB; p = 0.008), albumin (Alb; p = 0.027), globulins (Glb; p = 0.020), very-low-density lipoprotein cholesterol (VLDL-C; p < 0.001), gammaglutamyl transferase (GGT; p < 0.001), and high sensitivity C-reactive protein (hsCRP; p = 0.002), had an abnormal distribution. SCI: subdermal contraceptive implant; TG: triglycerides; HDL-C: high-density lipoprotein cholesterol; LDL-C: low-density lipoprotein cholesterol; AI: atherogenic index; DB: direct bilirubin; LDH: lactate dehydrogenase; and TP: total proteins. Kolmogorov Smirnov test was used to establishing the distribution of variables. Mann-Whitney U test and Wilcoxon test were used in order to analyze non-parametric values. Student t test and paired t tests were used for parametric variables.

**Table 3:** Metabolic variables before and after 4 months with the CSP

**Variable (RV)**	**Basal**	**4 months**	**p**	**Reference values**
Glucose [mg/dl]	89.5 (73.0-110.0)	92.2 (80.0-104.0)	0.289	65-114
Urea [mg/dl]	19.2 (8.6-35.1)	20.4 (12.8-28.8)	0.362	15-39
Creatinine [mg/dl]	0.55 (0.4-0.8)	0.59 (0.5-0.8)	0.071	0.5-1.2
Uric acid [mg/dl]	4.3 (2.8-6.5)	4.1 (3.1-6.1)	0.299	2.6-7.2
TG [mg/dl]	132.3 (50.7-307.0)	149.7 (82.3-224.8)	0.174	54-214
TC [mg/dl]	171.9 (142.1-222.3)	179.7 (137.8-235.7)	0.201	140-220
HDL-C [mg/dl]	41.8 (22.5-64.1)	44.4 (22.9-67.1)	0.220	21-75
LDL-C [mg/dl]	104.7 (58.9-143.5)	105.4 (73.1-152.1)	0.906	56-155
VLDL-C [mg/dl]	26.3 (10.1-62.0)	29.9 (16.5-45.0)	0.192	
AI	4.41 (2.9-8.4)	4.33 (2.9-7.6)	0.613	<4
TB [mg/dl]	0.64 (0.2-1.2)	0.51 (0.2-0.9)	**0.048**	0.2-1.0
DB [mg/dl]	0.09 (0.0-0.2)	0.09 (0.0-0.2)	0.797	0.0-0.2
IB [mg/dl]	0.55 (0.1-1.0)	0.42 (0.1-0.9)	**0.032**	
SGOT [U/l]	22.4 (15.6-37.5)	22.5 (15.0-46.3)	0.974	15-37
SGPT [U/l]	19.6 (8.0-46.8)	17.6 (9.6-38.8)	0.321	8-35
AP [U/l]	68.0 (42.7-98.8)*	60.4 (40.5-86.9)	**0.025**	37-110
LDH [U/l]	129.8 (80.6-166.8)*	120.8 (86.0-162.5)	0.053	106-274
GGT [U/l]	18.4 (9.6-31.9)	17.1 (5.6-34.5)	0.259	5-24
TP [g/dl]	7.0 (5.9-8.6)	6.9 (6.0-8.0)	0.431	6.7-8.2
Alb [g/dl]	4.1 (3.4-4.9)	3.9 (3.3-4.6)	0.061	3.8-5.1
Glb [g/dl]	2.94 (2.3-3.8)	2.98 (2.5-3.5)	0.721	

Results are expressed as median (percentiles: 2.5-97.5). Reference values (RV) are showed in the fifth column. Significant p values are shown in bold. Creatinine (p < 0.012), triglycerides (TG; p = 0.002), serum glutamic oxaloacetic transaminase (SGOT; p < 0.001), serum glutamic pyruvic transaminase (SGPT; p < 0.001), very-low-density lipoprotein cholesterol (VLDL-C; p = 0.002) and high sensitivity C-reactive protein (hsCRP; p < 0.001), had an abnormal distribution. CSP: contraceptive skin patch; TC: total cholesterol; HDL-C: high-density lipoprotein cholesterol; LDL-C: low-density lipoprotein cholesterol; AI: atherogenic index; DB: direct bilirubin; IB: indirect bilirubin; TB: total bilirubin; GGT: gammaglutamyl transferase; AP: alkaline phosphatase; LDH: lactate dehydrogenase; TP: total proteins; Alb: albumin; Glb: globulins. Kolmogorov Smirnov test was used to establishing the distribution of variables. Mann-Whitney U test and Wilcoxon test were used in order to analyze non-parametric values. Student t test and paired t tests were used for parametric variables.

**Table 4:** Thyroid screening, leptin, adiponectin, insulin, C peptide, IGF-1, and hsCRP before and after 4 months with SCI

**Variable**	**Basal**	**4 months**	**p**	**Reference values**
TSH (μU/ml)	2.0 (0.4-5.7)	1.6 (0.4-5.5)	**0.034**	0.34-5.60
T_4_ (μg/dl)	8.1 (4.2-11.7)	8.6 (6.3-12.7)	0.120	6.09-12.23
T_3_ (ng/dl)	120.6 (79.2-208.4)	123.1 (92.6-173.4)	0.622	75.0-250.0
FTI	7.8 (3.9-12.1)	8.3 (6.1-13.4)	0.367	4.5-15.0
PBI (μg/dl)	5.2 (2.7-7.6)	5.6 (4.1-8.2)	0.117	3.90-7.80
TU (%)	0.9 (0.9-1.0)	0.9 (0.8-1.0)	0.974	0.75-1.25
Anti-Tg (μU/ml)	15.8 (5.0-103.4)	14.2 (5.0-86.2)	0.625	5-100
Anti-TPO (μU/ml)	1.5 (0.3-59.0)	1.4 (0.3-43.0)	0.052	0-9
Insulin (μU/ml)	13.0 (4.5-43.1)	11.2 (3.7-26.8)	0.449	5-30
C peptide (ng/ml)	3.1 (1.3-7.4)	3.4 (1.8-5.8)	0.128	0.5-3.0
IGF-1 C (pg/ml)	251.2 (76.5-486.4)	279.7 (119.1-593.5)	**0.016**	115.0-307
Leptin (ng/ml)	9.1 (1.5-50.3)	11.1 (1.0-32.8)	0.812	4.1-22.4
Adiponectin (μg/ml)	16.0 (8.9-29.9)	14.7 (9.0-26.2)	0.837	3.9-25.6
hsCRP (mg/dl)	0.26 (0.02-1.2)	0.25 (0.03-1.0)	0.837	0.0-0.8

Results are expressed as median (percentiles: 2.5-97.5). Significant p values are shown in bold. All variables had an abnormal distribution (p < 0.050). TSH: thyroid-stimulating hormone; T4: total thyroxine; T3: total triiodothyronine; FTI: free thyroxine index; PBI: serum protein-bound iodine; TU: thyroid uptake; anti-Tg: anti-thyroglobulin antibodies; anti-TPO: anti-thyroid microsomal antibodies; IGF-1: insulin growth factor-1; hsCRP: high-sensitivity C-reactive protein. Kolmogorov Smirnov test was used to establishing the distribution of variables. Mann–Whitney U test and Wilcoxon test were used in order to analyze non-parametric values. Student t test and paired t tests were used for parametric variables.

**Table 5:** Thyroid screening, leptin, adiponectin, insulin, C peptide, IGF-1, and hsCRP before and after 4 months with the CSP

**Variable**	**Basal**	**4 months**	**p**	**Reference values**
TSH (μU/ml)	2.4 (0.8-6.2)	1.9 (0.7-3.9)	0.158	0.34-5.60
T_4_ (μg/dl)	8.5 (6.1-11.5)	9.2 (7.0-11.4)	0.072	6.09-12.23
T_3_ (ng/dl)	124.2 (68.1-188.3)	148.2 (110.0-220.2)	**0.001**	75-250
FTI	8.2 (5.7-11.9)	8.3 (6.9-10.0)	0.864	4.5-15.0
PBI (μg/dl)	5.5 (4.0-7.5)	6.0 (4.6-7.4)	0.070	3.90-7.80
TU (%)	1.0 (0.8-1.2)	0.8 (0.8-1.0)	**0.009**	0.75-1.25
Anti-Tg (μU/ml)	8.1 (5.0-333.1)	7.1 (5.1-230.4)	0.159	5-100
Anti-TPO (μU/ml)	0.7 (0.3-404.7)	0.7 (0.3-378.3)	0.915	0-9
Insulin (μU/ml)	8.7 (3.8-33.2)	9.2 (4.7-19.5)	0.547	5-30
C peptide (ng/ml)	2.7 (1.6-5.7)	2.8 (1.8- 4.2)	0.559	0.5-3.0
IGF-1 C (pg/ml)	227.0 (89.3-499.0)	225.4 (16.9-369.3)	0.775	115-307
Leptin (ng/ml)	9.5 (1.9-24.2)	8.9 (3.8-30.0)	0.090	4.1-22.4
Adiponectin (μg/ml)	16.6 (6.5-41.8)	17.9 (5.2-32.1)	0.409	3.9-25.6
hsCRP (mg/dl)	0.28 (0.01-1.0)	0.51 (0.1-1.3)	**0.013**	0.0-0.8

Results are expressed as median (percentiles: 2.5-97.5). Significant p values are shown in bold. With the exception of total thyroxine (T4; p = 0.373), thyroid uptake (TU; p = 0.748) and serum protein-bound iodine (PBI; p = 0.594), all variables had an abnormal distribution (p < 0.050). TSH: thyroid-stimulating hormone; T3: total triiodothyronine; FTI: free thyroxine index; anti-Tg: anti-thyroglobulin antibodies; anti-TPO: anti-thyroid microsomal antibodies; IGF-1: insulin growth factor-1; hsCRP: high-sensitivity C-reactive protein (hsCRP). Kolmogorov Smirnov test was used to establishing the distribution of variables. Mann-Whitney U test and Wilcoxon test were used to analyze non-parametric values. Student t test and paired t tests were used for parametric variables.

### Metabolic effects of contraceptive hormonal therapy

After the 4-month follow-up period, the pattern of metabolic changes induced by SCI in indigenous and mestizo women was quite similar. For example, glucose levels decreased 90.2-82.5 mg/dl (p = 0.002) in indigenous women and 89.6-84.1 mg/dl (p = 0.002) in mestizo women. TB and IB also significantly decreased only in indigenous women (p = 0.003 and p = 0.002, respectively). On the contrary, DB levels significantly increased 0.08–0.10 mg/dl (p = 0.046) in indigenous and 0.09-0.12 mg/dl (p = 0.007) in mestizo women. Although no modification in the lipid profile was observed in indigenous, in mestizo women there was a significant decrease in the levels of TG (148-100 mg/dl, p = 0.030), TC (163-139 mg/dl, p = 0.010), LDL-C (104-82 mg/dl, p = 0.035), and VLDL-C (29-20 mg/dl, p = 0.050) after the end of the study period. AI was lower in both groups after treatment, 3.9-3.3 (p = 0.009) for indigenous and 4.8-3.7 (p < 0.001) for mestizo women. Levels of SGOT and SGPT significantly decreased after the hormonal therapy period only in indigenous women: 29.1-23.0 IU/l (p = 0.004) and 28.3-21.9 IU/l (p = 0.047), respectively. A significant elevation in LDH (151.6-168.6 UI/l, p = 0.013), GGT (19.6-23.8 IU/l, p = 0.035), PT (6.9-7.3 g/dl, p = 0.029), and Alb (3.9-4.4 g/dl, p = 0.001) levels were observed only in mestizo women. Finally, in indigenous women we found a significant elevation in peptide C levels (2.7-3.9 ng/ml, p = 0.002), whereas a significant decrease in terms of insulin levels was found in mestizo women (15.0-10.4 μU/ml, p = 0.027). The only thyroid test significantly modified was TSH concentration, which decreased from 2.4-2.0 μU/mL (p = 0.035) in mestizo women. No significant changes were observed in the concentrations of adiponectin and leptin before and after treatment with CSP or SCI.

Because no one of the indigenous women used a CSP and due to the results observed for indigenous and mestizo women in the SCI group were similar, we performed the subsequent analysis of the results considering the women enrolled in the SCI group as a whole. Results for all variables analyzed are shown in Tables 2,3, 4 and 5. We found few significant changes in liver, renal, or thyroid tests. In some SCI users, we observed a decrease in the serum concentrations of glucose, TG, TC, LDL-C, and VLDL-C (Table 2). The percentage of users with an abnormal AI diminished from 75% at the beginning of the study to 41.6% after completing the follow-up. Liver function tests demonstrated a partial decrease in IB levels but an increase of 10 and 5 U/dl for LDH and GGT, respectively (Table 2). Also, we noticed an increase in IGF-1 levels (Table 4).

In the CSP group we observed a slight but significant decrease in TB, IB, AP, and TU levels as well as an increase in T_3_ (Tables 3 and 5). Also, we found an increase in hsCRP levels (Table 5).

## Discussion

Today it is well known that CSP has a similar tolerability and rate of effects as oral contraceptives [16]. For example, the CSP increases TC, HDL-C, TG, and decreases LDL-C [17]. The CSP also induce non-adverse effects on the carbohydrate metabolism and liver function tests [17]. Furthermore, it has been proven that the CSP may induce changes in the blood coagulation system [18,19]. Other common adverse effects include skin reactions at the site of application, breast discomfort during the first two cycles, and dysmenorrhea [20]. Nausea, emotional lability, headache, and occasional breakthrough bleeding are less frequently reported [21,22]. Effects of the SCI on most metabolic variables and on the blood coagulation system do not seem relevant [23-27]. Headache and dyspnea are frequent complaints [28], and a low rate of anxiety/depression (1-9%) has been reported [28]. Nausea, breast tenderness, lower abdominal pain, loss of libido, and fatigue are infrequent symptoms [28]. Weight gain has not consistently been associated with either CSP or SCI.

One hundred Mexican women were screened prior to entering a contraception program. In the CSP group, women were students, professionals, and housewives, whereas in the SCI group all users were homemakers. Despite these differences, the clinical characteristics were homogeneous between groups except for age because users of the CSP were younger.

Almost 30% of women were excluded from this study. Most were enrolled in the CSP group. We do not have a clear explanation for this finding. Perhaps the younger age of women in this group or the ease for withdrawal from the method determined this fact. A relatively low number of women were screened at the end of the 4-month study period in the CSP group. In the SCI group we were able to complete the study with 80% of the women originally enrolled. In this last group, only one user required withdrawal of the device 2 months after starting the study due to severe hemorrhage. After the 4-month study period, 30% of women in the SCI group had secondary effects.

Regarding laboratory abnormalities, the CSP slightly modified the metabolic variables analyzed but most of these changes were not clinically significant. Among the SCI users, changes were more evident but in some cases could be considered as beneficial. We observed a decrease in glucose concentration, a fact without a clear explanation. We may hypothesize that the increase of IGF-1 may be responsible for this phenomenon as previously suggested [29]. Indeed, in type 2 diabetic patients, IGF-1 glucose levels and insulin requirements are lower [30]. Moreover, in nondiabetic patients, IGF-1 increases insulin sensitivity and glucose metabolism while suppressing lipolysis and postprandial lipemia [31]. Although the increase in serum levels of IGF-1 may partially explain the changes in glucose metabolism, we must underline that this phenomenon does not appear to be a risk factor for contraceptive users.

On the other hand, the AI decreased and, of course, this is a finding that may suggest a positive relationship between the risk of atherothrombotic disease and the use of SCI. As described for the CSP, we also observed a significant decrease of IB. Previous studies evaluating the effects of SCI reported an increase of IB, hemoglobin, and hematocrit due to either the presence of amenorrhea or to the low frequency of hemorrhage [32-34]. In this study amenorrhea was infrequently observed but hemorrhage was a predominant symptom, a fact that may explain the decrease of IB levels. According to the thyroid and liver function tests, we observed minimal changes, only being significant for LDH, GGT, and TSH. These changes cannot be considered as adverse effects because the levels were always within normal ranges. Fortunately, this pattern of non-adverse effects remains similar when the SCI is used for longer periods of time [35].

Leptin and adiponectin, two cytokines that regulate metabolism, have gained attention in recent years due to their relation with other pathophysiological states. Low levels of adiponectin are associated with obesity, insulin resistance, and type 2 diabetes mellitus [36] as well as atherosclerosis, high blood pressure and coronary artery disease [37]. Moreover, a low adiponectin level is a strong risk factor for the development of metabolic syndrome [38]. On the other hand, leptin levels are not only associated with the amount of fat accumulated but also the energy balance of an individual. Abnormal leptin levels significantly influence the metabolic and hormonal processes in the organism [39]. High plasma levels of this cytokine are associated with obesity, insulin resistance, and glucose intolerance. These last two entities are strongly associated with the development of diabetes [40]. On these bases, we considered important to evaluate these cytokines before and after the treatment. However, we did not find significant changes in either CSI or CSP users. Adiponectin levels remained almost unchanged, whereas leptin showed minimal changes, especially in CSP users.

In order to determine if the metabolic changes may be related to the presence of an inflammatory state, we assayed the hsCRP, a very sensitive acute phase reactant for these purposes [41,42]. After 4 months, levels of this protein significantly rose only in the CSP group, up to almost 50% compared to basal levels. These data differ from previous reports in which hsCRP rose 220% after 2 months of SP use [18], a fact that suggests that the CSP may be associated with an important pro-inflammatory state only during the beginning of contraceptive therapy.

Due to the high mestizage of our population, we attempted to present a representative sample of Mexican women and to determine their overall metabolic response to contraceptive therapy. Although with modern techniques it is possible to establish the genetic background of an individual, it is accepted that sociocultural criteria and the presence of blood group O have sensitivity and specificity levels high enough to consider an individual as indigenous [15]. On the contrary, to identify a mestizo woman is quite simple after analyzing the phenotype of the individual and after interrogation about the origin of the user. To our knowledge, this is the first attempt to evaluate the metabolic effects of contraceptives in native-American populations. Eighteen indigenous women who were treated with SCI completed the study and the analysis of the metabolic changes induced by this contraceptive method showed that they had only slight differences as compared with mestizo women. Unfortunately, this analysis was not possible for the CSP group because none indigenous woman choose the CSP. Therefore, our results strongly suggest that at least the SCI does not induces clinical or biochemical relevant changes in either indigenous or mestizo Mexican women.

Of course, this study has some limitations. First, the non-randomized design of the research may be considered a major pitfall and a source of biased results however, considering that all around the world women chose the contraceptive method they desire to use, it was impossible to perform this study with a randomized design. Second, we are aware of our small sample a fact that may have some impact on the conclusions of the study. However, it must be stated that most of the literature addressing the secondary effects of contraceptive treatments include similar numbers of patients.

## Conclusion

Our results show that clinical symptoms are frequent in Mexican users of the CSP and SCI and that the symptomatology is sometimes clinically important. The increase in weight in half of the women was the predominant clinical symptom in users of both, CSP and SCI while abnormal uterine bleeding was highly frequent in users of SCI. Also, our study demonstrates that although estrogens or progestins may affect the overall metabolism of the users, these changes seem clinically irrelevant. It should be underlined that we followed the users only during a 4-month period, a fact that obligates us to exercise caution about the possible impact of these contraceptive methods for longer or perhaps shorter periods. Finally, it should be considered that we analyzed some metabolic patterns before and after the contraception therapy but induction of abnormalities affecting other physiological pathways cannot be ruled out.

## Abbreviations

CSP: Contraceptive skin patch; FDA: Food and drug administration; EE: Ethinylestradiol; NGMN: Norelgestromine; SCI: Subdermal contraceptive implant; ENG: Etonogestrel; DSG: Desogestrel; AC: Abdominal circumference; BMI: Body mass index; SBP: Systolic blood pressure; DBP: Diastolic blood pressure; AP: Alkaline phosphatase; LDH: Lactic dehydrogenase; SGPT: Serum glutamic pyruvic transaminase; SGOT: Serum glutamic oxaloacetic transaminase; GGT: Gamma glutamyl transpeptidase; TB: Total bilirubin; IB: Indirect bilirubin; DB: Direct bilirubin; TP: Total proteins; Alb: Albumin; Glb: Globulins; TG: Triglycerides; TC: Total cholesterol; HDL-C: High-density lipoprotein cholesterol; LDL-C: Low-density lipoprotein cholesterol; VLDL-C: Very-low-density lipoprotein cholesterol; TSH: Thyroid-stimulating hormone; T3: Total triiodothyronine; hsCRP: High-sensitivity C-reactive protein; IGF-1: Insulin growth factor-1; AI: Atherogenic index; T4: Total thyroxine; FTI: Free thyroxine index; PBI: Serum protein-bound iodine; TU: Thyroid uptake; anti-Tg: Anti-thyroglobulin antibodies; anti-TPO: Anti-thyroid microsomal antibodies.

## Competing interests

The authors declare that they have no competing interests.

## Authors’ contributions

JHJ: design, acquisition of data, analysis of blood samples and interpretation of data, drafting of the manuscript, final approval of the version to be published. EAGL: design, drafting the manuscript, final approval of the version to be published. MMH: acquisition of blood samples and clinical data, analysis of blood samples and interpretation of data, and drafting the manuscript, final approval of the version to be published. JFMP: first approach to the patients, acquisition of blood samples and clinical data, final approval of the version to be published. MARE: acquisition of clinical data, analysis and interpretation of data. GCH: acquisition of clinical data, analysis and interpretation of data. RJN: design, acquisition of clinical data, analysis and interpretation of clinical data, revising of the manuscript regarding intellectual content, final approval of the version to be published. XHG: first approach to the patients, acquisition of blood samples and clinical data, analysis and interpretation of data. RP-G: first approach to the patients, acquisition of blood samples and clinical data, analysis of data. II-S: analysis and interpretation of clinical data, revising of the manuscript regarding intellectual content, final approval of the version to be published. AM-C: design, analysis and interpretation of data, drafting of the manuscript, final approval of the version to be published. All authors read and approved the final manuscript.
